# Nurse staffing in large general hospitals in China: an observational study

**DOI:** 10.1186/s12960-020-0446-5

**Published:** 2020-01-17

**Authors:** Yuchi Shen, Weiyan Jian, Qiufen Zhu, Wei Li, Wenhan Shang, Li Yao

**Affiliations:** 1 School of Public Health, Peking University Health Science Center, Beijing, China; 2 Center for Health Policy and Technology Evaluation, Peking University Health Science Center, Beijing, China; 3 ZhongWei Institute of Nursing Information, Beijing, China; 4 Nursing Center, National Institute of Hospital Administration, Beijing, China

**Keywords:** Nurse to patient ratio, Staffing of nurses, Night care, Nursing

## Abstract

**Background:**

The appropriate staffing of nurses not only reflects the situation of nursing management of human resource, but also is related to the nursing quality in hospitals. This study investigated the staffing of nurses in large general hospitals in China.

**Methods:**

In this study, a database established by the National Centre for Nursing Care Quality Control, which conducted a national survey of the staffing of nurses in China mainland in 2017, was analysed. The time-point survey data of 20 375 departments in 668 large general hospitals in China were obtained, including the information of nurses and patients during the day (10:00 am) and at night (10:00 pm). Then, the staffing of nurses was evaluated by calculating the nurse to patient ratio (the average number of patients assigned to a nurse, NTP ratio). The Kruskal-Wallis test was performed to compare the NTP ratios during the day and at night among different regions and departments.

**Results:**

In large general hospitals, a nurse takes care of eight patients (NTP ratio = 1:8.0) during the day and 23 patients at night (NTP ratio = 1:23) on average. There were significant differences between day and night. In terms of different regions, a nurse in the hospitals in the western region takes care of 7.8 patients during the day (NTP ratio = 1:7.8) on average, and the nursing resource in the western region is more adequate than that in the eastern (1:8.0) and central (1:8.0) regions. At night, the eastern region has a higher level of NTP (1:23.0). In terms of departments, a nurse working in the ICU takes care of two patients during the day (NTP ratio = 1:2.0) and 2.9 patients at night (NTP ratio = 1:2.9). The level of NTP in the oncology department is relatively higher: 9.3 during the day and 34.0 at night. Other departments including internal medicine, surgery, obstetrics and gynaecology, paediatrics, and geriatrics have NTP ratios of 1:7–8 during the day and 1:18–25 at night.

**Conclusions:**

In China, the nurse staffing of large general hospitals has some regional and departmental patterns. The low level of nurse staffing at night may be a problem worthy of attention; the Chinese government needs to establish standards for different periods and departments to improve efficiency and quality of nursing.

## Background

In recent years, many countries in the world have achieved significant development in the human resources of health care [1, 2]; however, many issues are still prevalent, such as the shortage of health care professionals, unbalanced skill levels of health care workers [3, 4]. The nursing staff is an integral part of the health care workforce and the optimal staffing is a global interest [5, 6]. The World Health Organization (WHO) proposed a vision for health care [7]: accessible, acceptable, quality, and cost-effective health care with the staffing of nurses based on population needs. The nurse to patient ratio reflects the number of patients allocated to each nurse, the actual investment of human capital into patient care, and, to some extent, staffing of nurses. On the basis of the nurse to patient (NTP) ratio, the staffing of nurses is aligned with the goal of quality care to improve the quality of health care [8, 9].

Some studies showed that adequate nurses were closely related to the quality of care and patient safety. Aiken et al. [10] analysed the relationship between the staffing of nurses and patient mortality in nine European countries. For the increase of one patient per nurse, there was a 7% increase in the risk of death for patients hospitalized for 30 days [10]. Griffiths et al. [11] systematically reviewed the relationship between the staffing of nurses and patient outcomes. The results showed that the staffing of nurses was related to mortality, incidence of falls, length of hospital stay, and medication errors [11]. Chung and Sohn [12] studied the relationship between the staffing of nurses and in-hospital mortality of stroke patients in 615 hospitals in South Korea. The results showed that the staffing of nurses was a barrier factor in the mortality of stroke patients [12]. Zhu et al. [13] analysed the relationship between the staffing of nurses and patient outcomes in 181 hospitals in China. The results showed that when the nurse to patient ratio increased to 0.5~0.6, the outcomes of most patients were improved significantly [13]. To sum up, the rational allocation of nursing resources is an important factor to ensure the quality of nursing and improve the health of patients. The rational allocation of nursing resources not only helps to solve the main problems of nursing at present, but also provides the reference and support for the establishment of national applicable standards, so as to further guide the rationalization of staffing of nurses [14].

In China, the nursing workforce is rapidly growing while the NTP ratio is being gradually optimized. The first “Eleventh Five-Year” nursing plan was implemented in 2005 [15], which summarized the development status of nursing and set goals for nurse number in China. In 2012, the National Health Committee (formerly the Ministry of Health) issued the Guidelines on the Management of Hospital Staffing of Nurses, which states that nurses should be optimally staffed according to the workload, technical difficulty, and professional levels and that the average minimum NTP ratio should be 1:8 [16]. By the end of 2017, the number of registered nurses in China grew from 1.35 million (2005) to 3.8 million (2017), with an increase of 181.6% [17]. According to *The National Nursing Development Plan* (2016–2020), the total number of registered nurses is expected to reach 4.45 million [18]. In 2017, the China Care Quality Report showed that from 2014 to 2016, the overall median NTP ratio at large general hospitals was improved from 1:11.2 to 1:10.4 [16], which was an achievement of the previous efforts to develop and cultivate the nurse workforce. The increasing nursing workforce reflects the focus of the Chinese government in nursing in recent years. And in the meantime, the Chinese government also has higher requirements for the staffing of nurses in Chinese hospitals [19].

Tertiary A hospitals are the representative of large general hospital in China, which not only have the highest level of medical technology in China, but are also the major providers of critical care, as patients treated at tertiary A hospitals generally have more complex conditions [20], the problems of medical quality and patient safety [21] deserve more attention in these hospitals. According to the *National Care Nursing Development Plan (2016–2020)* [10], tertiary hospitals (bed to nurse ratio = 1:0.8) should assign more nurses to clinical work than secondary hospitals (bed to nurse ratio = 1:0.7) and primary hospitals (it depends). However, there are three shifts for nurses in Chinese hospitals, but standards have not established for different shifts. In addition, there is no systematic standard of nurse staffing based on NTP ratio in different levels of hospitals in China.

Some research shows that there is a difference between nurse staffing at night and during the day, and this difference may be related to nursing adverse events [22]. Therefore, in this study, we observe the NTP ratio during the day and at night. In addition, the demand for nursing varies from different departments, as the California area in the USA provided mandated nurse to patient ratios for different departments [7]. Therefore, this study will also observe the status of NTP ratio in different departments of large general hospitals in China.

In China, there are differences in the development among the eastern, central, and western regions. The eastern region has undergone rapid development with its regional GDP accounting for more than 50% of the total GDP of China, while the western region accounts for less than 20% [23, 24]. In terms of the distribution of health resources, the eastern region also has more than 1000 tertiary hospitals representing the highest level of medical care, and the number of tertiary hospitals in the eastern region is approximately equal to the sum of that in the central and western regions [25]. Therefore, our study also observed and described the NTP ratio from the regional level.

In this study, NTP ratio was used as the evaluation index for the staffing of nurses to analyse the data from 668 large Chinese general hospitals to evaluate (1) the overall NTP ratio, (2) NTP ratios during the day and at night, (3) differences in the NTP ratios between different departments, and (4) differences in the NTP ratios of the eastern, central, and western regions of China.

## Methods

### Design

The study employed a descriptive cross-sectional design to cover the eastern, central, and western regions of China.

### Setting and samples

For this cross-sectional study, a database established by the National Centre for Nursing Care Quality Control, which conducted a national survey of the staffing of nurses in China mainland in 2017, was analysed. The obtained data involved 20 375 nursing departments in 668 large general hospitals, covering 31 provincial-level administrative regions (excluding Hong Kong, Macao, and Taiwan) in China. The obtained information included the geographic information of the hospitals and the information of departments, such as the number of beds, nurses, and patients at the data collection point: during the day (10:00 am) and at night (10:00 pm).

In this study, the NTP ratio was chosen as the index to evaluate the level of staffing of nurses, because as a patient-oriented index, the NTP ratio can reflect the relationship between the demands for nurses and nursing resource [26]. The calculation of the NTP can reflect the number of patients directly assigned to each nurse and hence is a widely used evaluation index of staffing of nurses in other countries [27].

### Outcomes

Primary outcomes were the NTP ratios during the day and at night in large general hospitals in China, as well as the differences among different departments and among the eastern, central, and western regions of China. In this study, the NTP ratio = total number of patients in the ward/total number of registered nurses in a given period (the data collection point).

### Variables

The following data were used in this study: hospital types, hospital location (city and province), ward types, number of beds during the day and at night, number of patients in the department, and number of nurses in the department. The term “nurse” refers to fully qualified registered and professional nurses excluding intern/dispensing nurses, lead nurses, or head nurses.

The eastern, central, and western regions were included for the stratification analysis of the NTP ratio. The following departments were also included for the stratification analysis: internal medicine, surgery, ICU, paediatrics, obstetrics and gynaecology, oncology, and geriatrics [28].

### Statistical analysis

According to the Kolmogorov-Smirnov normality test, the number of nurses, patients, and beds did not conform to a normal distribution. Therefore, the NTP ratios across different departments were expressed as the median and upper and lower quartiles; the Kruskal-Wallis test was performed to compare the NTP ratios among different regions and departments. Stata v15 and Excel 2016 were used to compile and analyse the data.

### Ethics review

The approach taken to access and utilize the hospital data was analogous to the system of the National Health Service in England allowing researchers to access hospital statistics (HESonline). The data were released following a formal request to the National Centre for Nursing Care Quality Control, which assessed the risk and sensitivity of the request following normal internal procedures.

## Results

A total of 20 375 departments from 668 hospitals were included for analysis, including 303 hospitals in the eastern region, 190 in the central region, and 175 in the western region of China, accounting for 51.0%, 49.2%, and 54.2%, respectively, of all large general hospitals in each region.

The number of departments gradually decreased from the eastern region to the western region: a total of 9620 departments (47.2%) were from the eastern region, and 4488 departments (22.0%) were from the western region of China. On the departmental level, surgery and internal medicine accounted for about 67% of all departments, and geriatrics departments accounted for 2.2%. There were approximately 1400 to 1600 departments (7% to 8%) each for ICU, oncology, paediatrics, and obstetrics and gynaecology (Table 1).

**Table 1:** Characteristics of departments participating in the cross-sectional study

	*n* (%)
*N*	20 375 (100.0)
Region	
Eastern region	9 620 (47.2)
Central region	6 267 (30.8)
Western region	4 488 (22.0)
Department	
Internal medicine	6 615 (32.5)
Surgery	7 044 (34.6)
ICU	1 687 (8.3)
Oncology	1 495 (7.3)
Paediatrics	1 445 (7.1)
Geriatrics	457 (2.2)
Obstetrics and gynaecology	1 632 (8.0)

Table 2 shows the departmental data and daytime NTP ratio of the participating hospitals. The overall number of daytime beds in the departments was 44 (35, 54), the number of nurses was 5 (4, 7), and the number of patients was 42 (29, 54); thus, the NTP ratio was 1:8.0 (5.9, 10.3). In the western region, the number of beds and the number of inpatients were higher, and the number of nurses and the NTP ratio were similar to those at the national level. The numbers of beds and patients were the highest in oncology departments and were relatively low in ICU. On the other hand, the number of nurses was the highest in ICU. According to the Kruskal-Wallis test, a significant difference was observed in the NTP ratios across different departments: the NTP ratio in ICU was 1:2, the NTP ratio in oncology was about 1:9, the NTP ratio in paediatrics was about 1:7, and the NTP ratio in other types of department was about 1:8.

**Table 2:** Departmental data and daytime NTP ratio of the participating hospitals

	Beds, median (p25, p75)	Nurses, median (p25, p75)	Inpatients, median (p25, p75)	Patient/nurse		
				Median (p25, p75)	*P*	*χ*^2^
Total	44 (35, 54)	5 (4, 7)	42 (29, 54)	8.0 (5.9, 10.3)		
Region					< 0.001	18.68*
Eastern region	43 (34, 52)	5 (4,7)	41 (30,52)	8.0 (6.0,10.2)		
Central region	44 (34, 55)	5 (4, 7)	41 (28, 55)	8.0 (5.8, 10.7)		
Western region	47 (36, 58)	5 (4, 7)	44 (31, 57)	7.8 (5.7, 10.0)		
Department					< 0.001	4 443.04*
Internal medicine	47 (39, 57)	5 (4, 7)	44 (35, 56)	8.3 (6.6, 10.5)		
Surgery	45 (38, 54)	5 (4, 7)	44 (34, 55)	8.3 (6.5, 10.6)		
ICUs	16 (10, 22)	6 (4, 8)	12 (7, 19)	2.0 (1.5, 2.8)		
Oncology	50 (40, 60)	5 (4, 7)	52 (39, 66)	9.3 (7.4, 12.3)		
Paediatrics	42 (30, 55)	5 (4, 7)	39 (26, 53)	7.0 (4.9, 9.0)		
Geriatrics	38 (29, 50)	4 (3, 6)	35 (26, 46)	8.2 (6.0, 10.0)		
Obstetrics and gynaecology	41 (32, 51)	5 (4, 6)	39 (27, 51)	7.8 (5.7, 10.0)		

*Statistical significance

Patient/nurse = the number of patients/the number of nurses = 1/NTP ratio

Table 3 shows the departmental data and NTP ratios at night in the participating hospitals. The overall numbers of beds and patients in the departments were similar between day and night. The number of nurses at night was 2 (1, 2), which was significantly lower than that during the day. The NTP ratio was 1:23.0 (16.0, 32.0). In the eastern region, the NTP ratio was 1: 23.0 (15.5, 32.0), which was higher than that in the other regions. There were significant differences in the NTP ratios across different departments according to the Kruskal-Wallis test: the NTP ratio in ICU was about 1:3, the NTP ratio in oncology was 1:34, the NTP ratio in paediatrics was about 1:19, and the NTP ratio in other types of department was 1:21–24.

**Table 3:** Departmental data and night NTP ratios of the participating hospitals

	Beds, median (p25, p75)	Nurses, median (p25, p75)	Inpatients, median (p25, p75)	Patient/nurse		
				Median (p25, p75)	*P*	*χ*^2^
Total	44 (35, 54)	2 (1, 2)	42 (29, 54)	23.0 (16.0, 32.0)		
Region					0.012	8.74*
Eastern region	43 (34, 52)	2 (1, 2)	42 (29, 52)	23.0 (15.5, 32.0)		
Central region	44 (34, 55)	2 (1, 2)	41 (27, 55)	23.5 (15.5, 33.0)		
Western region	47 (36, 58)	2 (1, 2)	44 (31, 57)	23.3 (16.5, 32.0)		
Department					< 0.001	5 163.54*
Internal medicine	47 (39, 57)	2 (1, 2)	44 (34, 56)	24.5 (18.5, 33.5)		
Surgery	45 (38,54)	2 (1,2)	44 (33,55)	24.0 (18.0, 33.0)		
ICU	16 (10, 22)	2 (1, 2)	12 (7, 19)	2.9 (2.0, 4.0)		
Oncology	50 (40, 60)	2 (1, 2)	51 (39, 65)	34.0 (23.5, 50.0)		
Paediatrics	42 (30, 55)	2 (1, 2)	39 (27, 54)	18.7 (11.0, 28.0)		
Geriatrics	38 (29, 50)	2 (1, 2)	35 (26, 46)	23.5 (17.0, 31.0)		
Obstetrics and gynaecology	41 (32, 51)	2 (1, 2)	39 (27, 50)	21.0 (14.5, 28.0)		

*Statistical significance

Patient/nurse = the number of patients/the number of nurses = 1/NTP ratio

## Discussion

In China, the NTP ratio in large general hospitals is 1:8.0 during the day, which meets the requirements of the National Health Commission for the allocation of staffing of nurses, i.e. “a nurse should be responsible for no more than eight patients” [11]. In particular, there are some differences in the staffing of nurses among different regions. On the departmental level, ICU has a higher level of nursing resource (NTP = 1:2.0), while the level of nursing resource in the oncology department is relatively low (NTP = 1:9.3). There was no significant change in the number of patients at night, but the number of nurses was greatly decreased. On the regional level, the staffing level of nurses is relatively high in the east region and relatively low in the central region. On the departmental level, the ICU remains the most fully staffed department, with the same level of care of three patients per nurse at night. The NTP ratio in the oncology department was reduced to 1:34.0 (23.5, 50.0) at night, indicating that each nurse has to take care of 34 oncology patients at night.

Based on the results, certain regional and departmental differences were found in the staffing of nurses in large general hospitals in China. This may be related to the regional economic development, which impacts on recruit factors like the local pay and employment environment, the allocation of health resources, the value of labour force, the level of nursing needs, and other objective factors [29, 30]. In addition, it was found that the large general hospitals in China can basically meet the standard of staffing of nurses set by the government during the day [11], but the effects of the large drop in nursing manpower at night should be further analysed.

First of all, the majority of hospitalized patients will be in sleep at night, and hence, the nursing needs at night may be different from those during the day [31, 32]. From the perspective of hospital managers, it is reasonable to save part of nursing recourses for the day. For example, some regions of Australia establishing a mandatory NTP ratio also have different standards for the staffing of nurses between the day (1:4–5) and night (1:8–10) [33]. However, some mandatory NTP ratios, such as those in the USA, require the same staffing levels (about 1:4) in all shifts [34]. Secondly, several studies have shown that the quality of care is closely related to the optimal staffing of nurses throughout the day [35]. In addition, adverse events are more likely to occur at night [36]. Yin [37] analysed the distribution of 166 adverse events between day and night, and it was found that the incidence of adverse events reached the peak at night. These findings implied that fewer nurses are available at night and the reminding nurses are less alert [38, 39]. These results suggested that although it is difficult to determine the optimal level of night-time staffing of nurses, the large drop in nursing resources at night in large general hospitals in China may become a hidden risk to patient safety. At present, there is no national research on night-time staffing of nurses in China, and the related standards are scarce. However, since hospital managers do not have the motivation to strengthen nursing staffing at night, it may impair the quality of care by increasing the incidence of adverse events at night.

Judging from the growth rate of nurses in the past 10 years, the importance of nurse supplement has attracted the attention of the national government [9]. Further considerations should be paid to achieving the optimization of nurse staffing. At the same time, it is necessary to improve the quality of nursing. In order to improve the overall NTP ratio and to solve the nurse shortage problem at night, it is important to rely on scientific and reasonable measurements of nursing workload. To this end, it is necessary to carry out a large-scale comprehensive survey on staffing of nurses. At the same time, by studying the impact of staffing of nurses in different periods on patient safety, a standard of nursing resource allocation in different regions, periods, and departments suitable for the conditions of China should be established.

### Limitations

This study has some limitations. Due to the lack of individual patient data, it is impossible to analyse the effects of patient types on the staffing of nurses. Furthermore, because of the constraints in the data, the data from two time points on a specific day were used in this study to represent the levels of staffing of nurses during the day and at night rather than using the data of three shifts on several days.

## Conclusion

In general, there are some regional and departmental patterns in the staffing of nurses among large general hospitals in China. In addition, the low level of nursing staffs at night may be a problem that needs attention. In order to deal with this problem, the Government of China needs to establish standards for the staffing of nurses during different periods and in different departments, so as to guide hospitals to carry out rational allocation of nursing resource, enhance efficiency, improve nursing quality, and maintain patient safety.

## Data Availability

The datasets generated and/or analysed during the current study are not publicly available due there was not a part of the informed consent. However, the data are available from the corresponding author on reasonable request.

## Abbreviation

NTP: Nurse to patient
